# Socio-cultural determinants of antibiotic resistance: a qualitative study of Greeks’ attitudes, perceptions and values

**DOI:** 10.1186/s12889-022-13855-w

**Published:** 2022-07-28

**Authors:** Dimitrios Papadimou, Erik Malmqvist, Mirko Ancillotti

**Affiliations:** 1 The Sahlgrenska Academy, University of Gothenburg, Medicinaregatan 3, SE 413 90 Gothenburg, Sweden; 2 Centre for Antibiotic Resistance Research (CARe) and Department of Philosophy, Linguistics and Theory of Science, University of Gothenburg, Renströmsgatan 6, SE 41255 Gothenburg, Sweden; 3 Centre for Research Ethics and Bioethics, Department of Public Health and Caring Sciences, Uppsala University, Husargatan 3, SE-751 22 Uppsala, Sweden

**Keywords:** Antibiotic resistance, Antibiotic use, Qualitative research, Greece, Socio-cultural determinants of health, Social norms

## Abstract

**Background:**

Antibiotic resistance is a complex phenomenon heavily influenced by social, cultural, behavioural, and economic factors that lead to the misuse, overuse and abuse of antibiotics. Recent research has highlighted the role that norms and values can play for behaviours that contribute to resistance development, and for addressing such behaviours. Despite comparatively high antibiotic consumption in Greece, both at the community and healthcare level, Greeks have been shown to be relatively aware of the connection between antibiotic overuse and antibiotic resistance. This suggests that Greeks’ non-judicious use cannot simply be explained by lack of awareness but may relate to other factors specific to Greek society. The present study aimed to explore attitudes, perceived norms, and values in relation to antibiotics, in order to improve understanding of socio-cultural determinants of antibiotic resistance in Greece.

**Methods:**

Data were collected through online focus group discussions in 2021. Twenty Greeks were recruited through purposive sampling, aiming for as heterogeneous groups as possible regarding gender (12 women, 8 men), age (range 21–55, mean 33), and education level. Interview transcripts were analysed inductively using thematic content analysis.

**Results:**

Participants considered antibiotic overconsumption as a consolidated habit influenced by ease of access, social expectations and, more generally, cultural practices. While critical of such norms and practices, participants opposed stewardship measures that would prioritize the societal interest in maintaining antibiotic effectiveness over individual needs. Participants considered responsibility for antibiotic resistance to be shared by the whole society, but the role of government actors and health professionals as well as of food producers was emphasized. Notably, scepticism about the prospect of effectively managing antibiotic resistance in Greece was commonly expressed.

**Conclusions:**

The study makes explicit attitudes, perceived norms and values that, besides limited awareness, may contribute to non-judicious antibiotic use in Greece. These socio-cultural determinants of antibiotic resistance warrant further research and should be considered when designing measures aimed to mitigate this problem.

**Supplementary Information:**

The online version contains supplementary material available at 10.1186/s12889-022-13855-w.

## Background

Antibiotic resistance (ABR) enables bacteria to survive and proliferate despite exposure to antibiotic treatment. Resistance can be an innate feature of certain bacteria, or acquired through mutations in chromosomal genes or horizontal gene transfer [1]. ABR is an evolutionary process amplified by human action, as the use of antibiotics creates a selection pressure favouring its development in humans, animals, and the environment [2].

Worldwide, healthcare is already facing the effects of ABR; the diminishing effectiveness of antibiotics jeopardizes treatment of infections as well as prevention of complications to tertiary care associated with surgeries, cancer treatment, and other modern health procedures [3]. It has been estimated that about 1.3 million deaths globally were directly attributable to antibiotic resistant bacterial infections in 2019 [4]. In Europe alone, ABR accounted for approximately 33,000 attributable deaths and 875,000 DALYs (one DALY represents one lost year of “healthy” life) in 2015 [5].

The latest report on antibiotic consumption of the European Centre for Disease Prevention and Control ranked Greece the second highest country in EU/EES for total antibiotic use both at the community and healthcare level in 2020 [6]. Previous research has voiced concerns over limited knowledge about ABR among Greek prescribers and the general public, studied the effectiveness of information efforts targeting both these groups, and stressed the urgency for a national plan to improve regulations and guidelines on antibiotic prescription [7–9].

Although consistently regarded as a pre-requisite for judicious antibiotic use and for other behaviours that can impact ABR, information-giving in itself is often considered insufficient to change people’s behaviour [10, 11]. ABR is a complex phenomenon heavily influenced by social, cultural, behavioural, and economic factors that lead to the misuse, overuse and abuse of antibiotics. Such complexity has been acknowledged for decades [12], while at the same time social scientists have increasingly contributed to addressing this global challenge [13]. Recent research has also highlighted the role that the norms and values shared by a population can play for addressing ABR [14, 15]. One of the main lessons learned is that the design and feasibility of effective stewardship programs largely depends on the contexts to which they are applied [16]. Qualitative studies aimed at reaching deeper contextual understanding may therefore play an important role for efforts to decrease antibiotic use and address other ABR-related behaviours (e.g., food choices, vaccination, travelling).

In Greece, electronic prescription was legally mandated as the only antibiotic prescription option with the latest (2020) national plan against ABR [17]. This can surely be greeted as a step toward better prescription and dispensing of antibiotic treatments, as the lack of government regulation is known to be a major determinant of inappropriate practices [18]. Nonetheless, evidence suggests that social, cultural, and behavioural factors are essential for understanding antibiotic dispensing and community use [18]. Indeed, it is not surprising that despite consumption and misuse of antibiotics in Greece being above the EU average, Greeks have been shown to be relatively well aware of the connection between overuse and ABR [19]. Therefore, Greeks’ non-judicious use of antibiotics cannot be simply explained as a lack of awareness of ABR but may relate to other factors specific to Greek society.

The present study aimed to explore attitudes, perceived norms, and values in relation to antibiotics, in order to improve understanding of socio-cultural determinants of ABR in Greek society. Previous quantitative studies have focussed on the Greek population [9, 20–22], and Greek physicians [8, 9, 23–25], nurses [26], and pharmacists [27, 28]. However, to our knowledge, no previous qualitative research has been performed on the general population.

## Methods

### Design

A qualitative and explorative design was used to collect data through focus group discussions (FGDs). FGDs provide insight into behaviour by generating a process that helps participants to self-disclose and are particularly useful to explore a group’s norms as well as the range of viewpoints that exist within a population [29, 30]. This study was designed and conducted in accordance with good practice guidelines, including recommendations for virtual focus groups during a pandemic [30, 31].

The semi-structured interview guide was adapted from Ancillotti et al. [32] after a thorough discussion within the research team and a pilot test. The original interview guide was constructed using the Health Belief Model as methodological framework [32]. However, the interview guide proved to be useful also in generating contents connected to the value and social dimensions of ABR, worth being investigated in a secondary analysis [15]. The interview guide was translated and adjusted so that meetings would not exceed one-hour duration. Three less generative questions were eliminated, and the transition question and one key question were transformed into detailed probing questions – i.e., questions designed to promote elaboration on specific topics when deemed relevant (see Additional file 1).

Consolidated criteria for reporting qualitative studies (COREQ) were adhered to (see Additional file 2).

### Sampling, recruitment & data collection

Participants were recruited from the Greek general population. Inclusion criteria: aged over 18 years, proficient in Greek, and Greek citizenship and residency. Exclusion criteria: relevant healthcare education or health-related occupation. The rationale for these exclusion criteria was to minimize the impact of any individual’s authority on group dynamics. Participants were recruited by the first author in January 2021 using a social media platform (Facebook) through purposive sampling, aiming for the composition of a sample as heterogeneous as possible regarding gender, age, and education level. Even before the COVID-19 pandemic limited the possibility of face-to-face encounters with participants, social media platforms and online video platforms (such as Zoom) had been recognised as valuable tools for recruitment and data collection in health research [33].

The FGDs were conducted by the first author, a male student of Public Health Science (MSc) with a background in Molecular Biology and Genetics (BSc), using Zoom in February 2021. Participants were informed about the topic of the discussion. They had no prior relationship with the interviewers nor among themselves. The FGDs lasted between 45 and 60 minutes. After about 10 minutes, participants watched a six-minute animated video presenting basic facts about ABR mechanisms, how antibiotics work, and global issues connected to access, excess, and antibiotic pollution [34]. Data saturation, i.e. when new data tend to be redundant in view of data already collected [35], was reached after three FGDs. A fourth FGD was held for verification. The interviews were audio recorded and transcribed verbatim. No dropouts occurred. Participants received no compensation for their participation.

### Data analysis

The transcripts were coded manually and analysed inductively using thematic content analysis [36]. In the first stage, the first author, who is a Greek native speaker, coded the transcripts and discussed the process, interpretation issues, and preliminary findings multiple times with the rest of the research team, which, in addition to the first author, included an Associate Professor of Medical Ethics (second author) and a Postdoctoral researcher with a PhD in Medical Science (last author). To become familiar with the content, the coder read the transcripts multiple times while starting the open coding: the process of identifying themes and categories emerging from the text and taking note of words and phrases that could sum up relevant content.

The second stage meant eliminating duplications and overlapping or too similar categories and was carried out by the whole research team. In the final stage, distinctions were refined by sorting the remaining categories into groups. The research team discussed the results to find consensus. The categories are descriptive and use the participants’ terms. The themes are the result of a final abstraction process and are thus interpretative. Transcripts were not returned to participants, who were not further involved after the FGDs.

## Results

Twenty members of the general public participated in four FGDs (see Table 1).

**Table 1:** Demographic information for the 20 participants

	G1	G2	G3	G4	Total
Gender					
Woman	4	2	4	2	12
Man	0	3	2	3	8
Age range					
Minimum					21
Mean					33
Maximum					55
Location					
North (Macedonia & Aegean islands)					12
West (Epirus)					2
East (Thrace)					2
Central (Attica)					2
South (Peloponnese & Crete)					2
Education level					
High school					2
Bachelor’s degree					11
Master’s degree					7

Five themes emerged from the analysis: norms, values, responsibility, scepticism, and alternative practices (see Table 2).

**Table 2:** Overview of themes and categories and their relative exemplar quotes

Theme	Category	Quote
Norms	Social influence	Q1, 2
	Ease of access	Q3
	Cultural habits	Q4
Values	Human life	Q5, 6
	Altruism	Q7
	Emotions in decision-making	Q8
Responsibility	Individual	Q9
	State	Q10
	Collective	Q11, 12
	Physicians	Q13
	Patients	Q14
Scepticism	Corruption	Q15
	Food production	Q16
Alternative practices	Preventive measures	Q17
	Alternative treatments	Q18

### Norms

Under ‘norms’ are categorized views expressed by the participants about the rules or standards of behaviour that they consider established in their society and internalized, influencing people’s thoughts, feelings and behaviour.

Participants looked upon antibiotic overconsumption as a consolidated practice in Greece. Individuals’ social circle but also physicians were referred to as exerting considerable influence towards excessive use of antibiotics:

(Q1) Everyone takes antibiotics like caramels and turns to antibiotics without necessarily visiting a physician to prescribe them, just because a person took the same antibiotic when he/she had the same symptoms, and therefore this will do the same for me. (Group(G)2 Woman(W)3)

(Q2) And sometimes even physicians are telling you to take an antibiotic and you will be fine. So … doctors also drive you to this pathway. (G2W2)

Participants considered it a major problem that antibiotics are easily accessible and that unduly obtaining antibiotics is not hindered significantly by either cost or the prescription procedure:

(Q3) I also think the problem is the rather easy access, because of the cost and because no serious prescription is required. (G2W1)

Participants explicitly mentioned that the Greek culture may be considered the main antagonist to engagement in behaviour beneficial to the greater good:

(Q4) Unfortunately, it is deeply rooted inside our culture, the Greek culture that antibiotics are used as a precaution … What matters is to fight against a culture that is deeply rooted inside us. And even now that prescription is more controlled, antibiotics will be given with abandon. (G1W3)

### Values

Under ‘values’ are categorized general, abstract views expressed by the participants about what is important and what should matter in relation to ABR.

Participants were critical of the idea of withholding antibiotic treatment to prioritize the societal interest in maintaining antibiotic effectiveness over individual needs. In doing so, they often supported their arguments by referring to the role of sentimental and cultural values as well as to inalienable individual rights. Respondents sympathized with each individual who would need antibiotic treatment and emphasized the equal value of all human life:

(Q5) I believe that human life, whether it concerns an individual or millions, has the same value … I do not think that many will agree to strict measures. (G2W1)

As a consequence, they regarded it as morally questionable to limit individuals’ access to potentially beneficial treatment in the name of the greater good:

(Q6) We cannot say that a part of society will suffer for the good of the whole society. Because if parts of society suffer, society can be dissolved. If the patient will certainly survive but will suffer for the good of all the others, in the end, who is responsible for making this decision? Who has the right to say that you will suffer for the good of others? (G2Man(M)1)

Nonetheless, a few participants claimed that they would forego antibiotics for themselves and close ones, as long as this would not put their life in danger:

(Q7) [When it is not a life-threatening infection], I would avoid using antibiotics for myself and the people around me. (G1W3)

Elaborating on the values underpinning cultural norms, the role of emotions in decision making, including resource allocation, was emphasized:

(Q8) I just think that I could not stand it personally and emotionally because every human being is important to the society in which he/she lives. It may have a logic to help the common good, but if the person in danger was one of my close people, I would be against it. So, I believe that we Greeks work more with emotion than with logic. (G2W4)

### Responsibility

Under ‘responsibility’ are categorized participants’ views about who is responsible for ABR, what such responsibility entails, and where blame should be directed.

Participants held every individual responsible for ABR based on the idea that everyone contributes to it:

(Q9) Obviously we have a personal responsibility. Antibiotics are needed in our lives … It depends on how somebody wants to overcome an illness and how much one wants to suffer. It is a personal matter if I take antibiotics. No one obliges me in the end. The state is not responsible for my reckless use. (G2W2)

However, for the individual to act responsibly there would be a need for the state to set conditions conducive to individual responsibility:

(Q10) I focus on our personal responsibility but also on the state that must set the limits and then, certainly, individual responsibility. I believe that first the state must function properly. (G2W4)

The notion of collective responsibility was a vague concept for most of the participants: a scenario in which society would share and act on the idea of a common purpose did not seem realistic:

(Q11) So our society does not have the [features] to deal with it. Beyond antibiotics, there is a public confusion, in general. We have lost track of things. (G4M1)

In contrast, a couple of participants grasped the notion of collective responsibility and highlighted its centrality:

(Q12) I only see it as collective. My individual practice is not enough. Resistance is something that happens and is transmitted by all social groups. In a globalized society and economy where people travel, you cannot talk only about individual actions, subjectively leading to relative results. So obviously the society must make judicious antibiotic use because overconsumption is creating the problem. (G3M3)

Furthermore, physicians were held to have a special responsibility because of their profession. Patients were also considered to have their share of responsibility, especially in the prescribing process:

(Q13) When we do use and not misuse, we have a better result. Now to what extent can I be responsible for another patient? I believe that the issue is purely a matter of physician responsibility because each of us individually cannot make such a decision. (G3M2)

(Q14) You cannot force a prescription on an adult. If antibiotics are a necessity, then both the patient and the physician should be really responsible. (G1W4)

### Scepticism

Under ‘scepticism’ are categorized participants’ expressions of disbelief about the prospects of effectively managing ABR.

Corruption in the Greek healthcare system was regarded as an obstacle to the adaption of measures for the mitigation of ABR in the country:

(Q15) The mindset towards antibiotics should change but unfortunately, that is not feasible for Greece, I am referring to the corruption in the health system. (G4M1)

Some participants voiced the concern that the use of antibiotics in meat and dairy production is uncontrollable, actually overshadowing human medical use:

(Q16) I was surprised that there are animals in big farms crammed and fed with antibiotics. I did not expect it, personal use is possible to be controlled, but I think you cannot control this. Therefore, possibly this is the biggest problem. (G4M3)

### Alternative practices

Under ‘alternative practices’ are categorized specific attitudes towards antibiotic treatment of participants who expressed their preference for alternatives to it.

It was suggested that alternative health practices exist, which help preventing or coping with bacterial infections and avoiding antibiotics:

(Q17) I looked it up on my own and found health practices that daily help me to deal with the health problems I have/had. I'm trying not to get to the point of taking antibiotics for an infection. (G4W1)

The use of other medical remedies but also homeopathic pills was mentioned as preferred over antibiotics, which were considered as the final option:

(Q18) I use homeopathic pills for my children and myself. Antibiotics are my last resort. (G2W4)

## Discussion

Against the background of comparatively high levels of antibiotic use in Greece and limited understanding of related socio-cultural factors, we explored Greeks’ attitudes, perceived norms, and values regarding antibiotics and ABR. While previous research has emphasized ignorance as a major driver of improper antibiotic use in Greece and recommended informing prescribers and the public in response [7–9], our findings point towards other challenges. In the following, attitudes, norms, and values potentially acting as socio-cultural determinants of ABR in the Greek society are discussed.

In general, participants recognized the severity of ABR, but stressed the complexity and difficulty of effectively mitigating it. They were sensitive to the tension between individuals’ interests and the common good that is often considered central to ABR mitigation [37, 38], generally expressing stronger priority to the individual than found in studies from other contexts [14, 32, 39]. Participants were aware of individuals’ potential contribution to ABR and acknowledged that individuals have a role in mitigating it. However, relying on individual responsibility alone was considered a fragile strategy due to individuals’ susceptibility to social pressure and emotional decision-making. The latter was conceptualized as a Greek cultural characteristic potentially partly determining non-judicious antibiotic use, linking to previous research connecting culture and emotion [14, 40]. Moreover, participants found individuals’ responsibilities to be conditioned upon those of state actors and physicians. The belief that these latter actors fail in their duties, e.g., due to corruption and systematic over-prescription, reinforced participants’ doubts about the prospects of effectively tackling ABR, affirming the connection between corruption and higher antibiotic consumption rates also found by other researchers [41]. This negative perception could be interpreted as lack of trust in healthcare governance and practice. Trust can be considered as a determinant of ABR, in general as a determinant of health and social cooperation [42, 43], and in particular as a factor known to be linked with willingness to contribute to antibiotic stewardship, for instance by postponing antibiotic treatment [44] and accepting doctors’ decisions not to prescribe antibiotics [45]. While these findings point to the importance of strengthening public trust in healthcare institutions for effective ABR management and stewardship in Greece, they also make explicit disbelief concerning lay people’s capacity to contribute to curbing ABR. Previous studies have highlighted the perils of public scepticism about the possibility of improving the current ABR situation [32, 46]. Whether linked to lack of trust, personal detachment, egoism or perceived low self-efficacy of engaging in judicious behaviour, such scepticism represents a potential obstacle to the promotion of a more sustainable use of antibiotics in Greek society.

Some participants mentioned resorting to alternative health practices. This may be due to anti-medicine or anti-establishment convictions [47, 48] or, more generally, be a token of participants’ lack of trust in the healthcare system. However, in some cases, it could also signal understanding of the importance of antibiotics and readiness to engage in judicious behaviours in relation to antibiotic use and ABR in general, including by adopting infection prevention measures. Potentially supporting the latter interpretation, cultural values such as concern for the biosphere and for naturalness have been positively associated with awareness of ABR risks [46]. Given the significance of trust for effective antibiotic stewardship and the potential risks of relying on complementary and alternative medicines, these phenomena, and the complex relation between them, warrant further investigation in a Greek setting.

Some comparisons can be made with similar studies performed in other countries with relatively high rates of antibiotic use and antibiotic resistance in the WHO European region [49]. The centrality of trust in health professionals and health institutions as a factor promoting a judicious use of antibiotics – and conversely, lack of trust as a hindering factor – was emphasized by recent research on the Spanish, Turkish, and French public [50–52]. In contrast to the findings of the present study, participants in focus groups in Spain showed a relative lack of knowledge and a lack of perception of the problem of antibiotic resistance [50]. The normative role of individuals’ social circle, mainly friends and families, was recognized also by Westerling et al. [51]. With regards to the notion of responsibility for ABR, a notable difference was detected with the findings from Essilini et al. [52]. The French focus group participants did not perceive ABR as a personal responsibility.

In comparison to previous studies performed in Sweden (the interview guide used in this study was adapted from the Swedish research) [15, 32], some commonalities and differences stand out, especially about the notion of responsibility. Both Swedish and Greek participants expressed the belief that there is a personal responsibility for ABR. Where they clearly diverged was in their consideration of the collective dimension of responsibility. While the Swedish participants considered the decreasing availability of effective antibiotics a problem of justice prompting both individual and collective responsibility for ABR [15], the notion of collective responsibility was a vague concept for most of the Greek participants, who tended to see as unrealistic a scenario in which society would share and act on the idea of a common purpose. Moreover, in contrast to the Greek participants, the Swedish participants expressed high levels of trust in institutions and in their individual self-efficacy to engage in judicious behaviours in relation to ABR [32].

This study reinforces the growing recognition of the importance of norms and values for antibiotic use and related mitigation strategies [14, 15] and of the need to consider contextual variation in this regard [16]. Recent literature has stressed that, amongst other challenges, ABR raises serious ethical ones, which require justifiable ethical principles to be formulated and applied [37, 53, 54]. In addition, however, people’s actual normative and evaluative commitments, such as those found in this study, need to be understood to ensure feasibility of policies based on such principles [38, 55]. In terms of concrete measures tackling specific obstacles, lack of trust in healthcare institutions and perceived low individual self-efficacy, if confirmed in quantitative investigations, could be addressed in adequately framed communication campaigns as well as in doctor-patient communication, as suggested also by other research [32, 51, 52].

Some limitations should be noted. In general, the results from a qualitative study are not strictly speaking representative of the studied population as a whole. Moreover, over half of the participants were from Northern Greece, most had received university-level education, and the oldest was 55 years. It is possible that a more heterogenous sample would have yielded more nuanced data, potentially affecting the relative prominence of certain themes. In addition, the focus group design together with the online format may have limited some participants’ willingness to interact and communicate. However, such effects were counteracted by limiting group size and posing probing questions. In addition to the relative homogeneity of the sample and the online setting, the structure of the interview guide and the informational video shown 10 minutes into discussions may have contributed to delimit the space within which the discussions happened, partly explaining the relatively early data saturation. The fact that FGDs took place during the COVID-19 pandemic may have affected the views expressed by the participants, in particular their disbelief about the prospects for effectively curbing ABR.

## Conclusions

This study highlights a complex set of attitudes, perceived norms and values that, besides limited knowledge, potentially contribute to non-judicious antibiotic use in Greek society. These include an experienced social expectation to retain inappropriate practices, reluctance to accept the imposition of burdens on individuals in the pursuit of collective goals, scepticism about the effectiveness of individual efforts in a context where institutional actors are seen as shirking their responsibilities, and a willingness to engage in alternative health practices. Future research, including quantitative studies, is needed to gain a more detailed understanding of the role of these and other socio-cultural determinants of antibiotic resistance in Greece and to design effective, contextually sensitive mitigation measures.

## Supplementary Information

**Additional file 1.** Interview guide.

**Additional file 2.** COREQ

## Data Availability

The datasets generated and analysed during the current study are not publicly available in order to preserve pseudonymity of study participants but are available from the corresponding author on reasonable request.

## Abbreviations

ABR: Antibiotic resistance
FGD: Focus group discussion
G: Group
M: Man
Q: Quote
W: Woman
