# Supplementary material for: Socio-cultural determinants of antibiotic resistance: a qualitative study of Greeks’ attitudes, perceptions and values
Source: BMC Public Health. 2022 Jul 28;22:1439. doi: 10.1186/s12889-022-13855-w (PMC9333897; doi:10.1186/s12889-022-13855-w)
Supplement: Supplementary file 1 — Additional file 1. Interview guide. [file 12889_2022_13855_MOESM1_ESM.docx]

## Additional file 1. Interview guide

| **Question type** | **Question** | **Probing Question** |
| --- | --- | --- |
| Opening | 1. What is your name and why did you decide to partake in this discussion? |  |
| Introductory | 2. What is the first thing that comes up in your mind about antibiotics? | What is your experience with antibiotics? |
| **- Break and short movie (Transition) -** | | |
| Key | 3. As you saw in the movie, there are risks related to antibiotic resistance. What do you think about it and why? |  |
|  | 4. Thanks to antibiotics, healthcare has had great success in treating infections. We know that increased resistance is of particular concern for groups at risk, such as immunocompromised patients, those who undergo major surgery, patients in cancer treatment, etc. For their sake, it is important that we all use antibiotics responsibly. What do you think about this? | Group reflection about whether responsible use is burdensome, about who is or should be held responsible and about future generations. |
|  | 5. Some researchers said: “The solution may ultimately require us to put society before the individual. That is, halting the rise of resistance may only be achievable if some patients go untreated”. Is this reasonable? |  |
|  | 6. What are you prepared to do to counter antibiotic resistance? | Group reflection about whether they could improve their behaviour concerning vaccines and travelling  How would you react if you had a fever and cough, and the physician says no to prescribe antibiotics to you?  As you saw in the movie, tons of antibiotics are used to produce meat. Do you think that we should revise the way we buy food, what we eat to keep antibiotics working? You know, also for the future. |
